# The Landscape of Integrated Domains of Angiosperm NLR Genes Reveals Continuous Architecture Evolution of Plant Intracellular Immune Receptors

**DOI:** 10.3390/plants15010081

**Published:** 2025-12-26

**Authors:** Zhen Zeng, Sai-Xi Li, Wen-Shen Wu, Peng Zhao, Zhu-Qing Shao, Yang Liu

**Affiliations:** School of Life Sciences, Nanjing University, Nanjing 210023, China; zengzhen561@163.com (Z.Z.); 602024300020@smail.nju.edu.cn (S.-X.L.); wenshenwu@smail.nju.edu.cn (W.-S.W.); zhaopeng33@smail.nju.edu.cn (P.Z.)

**Keywords:** plant immune receptor, NLR gene, integrated domain, angiosperm, effectors

## Abstract

Nucleotide-binding site-leucine-rich repeat (NLR) proteins are key intracellular immune receptors in plants. Integrated domains (IDs) can occasionally be fused with NLRs, contributing to their functional diversity. However, the diversity and evolutionary patterns of NLR-IDs across angiosperms remain poorly understood. In this study, we analyzed 305 angiosperm genomes and found that the proportion of NLR genes containing IDs (NLR-ID genes) ranges from 0% to 38.3%, with an average of 10.6%. A total of 1226 unique IDs were identified, some of which are widely distributed, while others are specific to particular taxa. Notably, 415 of these IDs are homologous to plant proteins targeted by pathogen effectors, suggesting their role as candidate decoys. Comparative analysis of NLR-IDs in two subfamilies—TIR-NLR (TNL) and CC-NLR (CNL)—revealed that TNL genes have a significantly higher frequency of IDs, with the C-JID and DUF3542 domains being most prevalent. N-terminal fusion of the DUF3542 domain in CNL genes correlates with the loss of the MADA motif. Our findings expand the understanding of NLR-ID diversity and provide insights into the dynamic evolution of NLR protein architecture in angiosperms.

## 1. Introduction

Plants deploy cell surface and intracellular immune receptors to detect pathogen-derived molecules and initiate defense responses [1,2]. Nucleotide-binding site-leucine-rich repeat (NBS-LRR or NLR) proteins, which originated in the common ancestor of green plants, constitute the major type of intracellular immune receptor of plants [2,3,4,5,6]. Canonical NLR proteins contain a C-terminal leucine-rich repeat (LRR) domain, a central nucleotide-binding site (NBS) and an N-terminal variable domain [7]. On the basis of three different N-terminal domains, namely coiled-coil (CC), Toll/Interleukin-1 receptor (TIR), and resistance to powdery mildew 8 (RPW8), angiosperm NLR proteins are divided into three subclasses: CC-NLR (CNL), TIR-NLR (TNL) and RPW8-NLR (RNL) [8]. A large number of CNL and TNL proteins function by detecting effectors released into plant cells from specific pathogens and are therefore termed sensor NLRs [1,2,9]. In contrast, two RNL lineages (ADR1 and NRG1)-which diverged in the common ancestor of angiosperms-mainly encode proteins involved in the signal transduction of sensor NLRs, and thus serve as core helper NLRs [7,10].

Sensor NLRs can recognize effectors from pathogens through direct physical binding or by monitoring the modification of plant proteins by effectors [2]. Host proteins monitored by sensor NLR proteins are called guardees or decoys to suggest their roles as effector-targeted targets or mimics of effector-targeted targets, respectively [11]. Some of these decoys can be fused to NLR proteins as discrete structural domains, which are referred to as a type of integrated domains (IDs); NLR genes harboring integrated domains are correspondingly designated as NLR-IDs [12,13]. Several intensively investigated NLR-ID genes fit the integrated decoy model, including the *Arabidopsis thaliana* TNL protein RRS1, which fuses the WRKY domain as an ID, and the *Oryza sativa* CNL proteins RGA5 and Pik-1, which both integrate a heavy metal-associated (HMA) domain [14,15,16,17,18]. Both of these IDs have been reported as decoys of cognate effectors of different pathogens.

During the arms race between pathogens and plants, many effectors evolve to suppress plant defense by targeting key immune factors [3] and facilitate infection by interacting with susceptibility factors. In the integrated decoy model, some IDs are mimics of effector targets. Therefore, the presence of a specific ID in the NLR-ID gene strongly implies that the nonintegrated ID homologs may be involved in plant immunity or other pathogen-related processes. Inspired by this, a ZBED protein from rice, which contains three BED domains that present as an ID of a CNL gene against *Xanthomonas oryzae*, was identified as a novel immunity-related factor [19]. More recently, several genes encoding the HMA domain were identified as susceptibility genes in both rice and *Arabidopsis* [20,21]. These examples suggest that exploring IDs in the NLR genes provides an alternative way to identify novel effector target proteins involved in plant immunity.

Genome-wide analysis of NLR-ID genes by several recent studies revealed over two hundred IDs from dozens of plant genomes [13,19,22,23]. While a small number of cross-species conserved IDs have been observed, the frequently detected species-specific IDs suggest that the repertoire of ID diversity is far from being thoroughly recognized, particularly due to the limited taxonomic breadth of previous sampling. On the other hand, despite the identification of more than two hundred IDs [13,18,24,25], few studies have investigated these IDs in the context of effector interactomes to explore their potential roles in decoy functions or identify candidate effector-interacting proteins. Additionally, the evolutionary connections between ID fusion and functional structural features of NLR proteins—such as the MADA motif, a functionally critical element for CNL-mediated immunity—remain largely unaddressed. Recently, an angiosperm NLR atlas was established, which collected over 90,000 NLR genes from more than 300 genomes across diverse angiosperm lineages [26]. The dataset provides a unique opportunity for deeply exploring the abundance, diversity and dynamic evolution of angiosperm NLR IDs. Furthermore, large-scale protein interaction analyses between effectors from different pathogens and plants have been carried out in several studies, which can serve as a resource to elucidate the potential roles of IDs [27,28,29].

Here, we performed a comprehensive investigation into the landscape of the IDs of NLR genes in 305 angiosperms. By aligning identified IDs to effector-interacting protein resources, we evaluated their potential roles as effector decoys. Phylogenetic distribution analysis of identified IDs elucidated the dynamic evolution of diverse IDs. Detailed evolutionary analysis was conducted on the most prevalent IDs identified from two sensor NLR subclasses. This comprehensive angiosperm-wide study of NLR IDs can help better understand the architectural evolution and functional innovation of plant NLR genes.

## 2. Results

### 2.1. ID Integration Is Prevalent Among Different Angiosperm Species and NLR Subclasses

**Figure 1:**
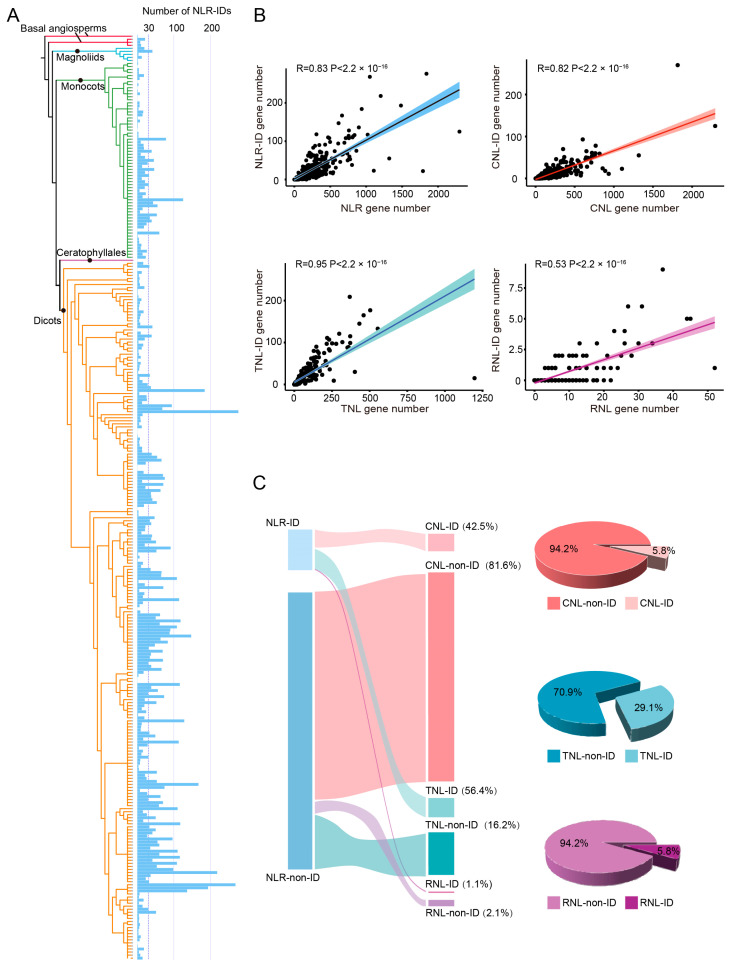
NLR-ID gene content variation among angiosperm genomes. (**A**) The phylogenetic relationships of the 305 analyzed species were constructed according to the APG IV system [30]. The NLR-ID gene number for each species is shown in the blue column. Different colored branches represent different plant lineages: red represent Basal angiosperms, blue represent Magnoliids, green represent Monocots, orange represent Dicots. (**B**) Spearman correlation analysis for gene numbers between NLR-ID (or NLR-ID subclasses) and NLR (or NLR subclasses) in each species. Dots represent individual species; Lines represent the fitting trend of the correlation; Colors correspond to NLR subclasses (blue for TNL, red for CNL, purple for RNL). Sample size (*n* = 305), corrected threshold = 0.0125, FWER ≤ 0.05. (**C**) Proportion of NLR-ID relative to the total NLR gene or different NLR subclasses. NLR-ID: NLR genes with integrated domains; NLR-non-ID: NLR genes without integrated domains.

To delineate the distribution of ID-containing NLR genes within angiosperm genomes, we conducted an extensive analysis of 91291 NLRs retrieved from the BIG database (PRJCA005581) [26], encompassing 305 angiosperm genomes across diverse angiosperm lineages (Figure 1A). Among these, 9651 NLRs from 286 genomes were found to have integrated at least one ID within their protein sequences, constituting approximately 10.6% of the NLRs examined (Table S1). The frequency of NLR-ID genes varied dramatically across angiosperm genomes, ranging from one in *Amborella trichopoda*, *Persea americana* and *Oropetium thomaeum* to 276 in *Coffea arabica* (Figure 1A and Table S2). The top ten species, with 125 to 276 NLR-ID genes, included a monocot (*Triticum aestivum*) and diverse eudicots from six different orders, namely Gentianales (*Coffea arabica*), Asterales (*Chrysanthemum seticuspe*), Fabales (*Abrus precatorius* and *Arachis hypogaea*), Rosales (*Rosa multiflora*), Brassicales (*Camelina sativa*) and Fagales (*Quercus lobata*, *Quercus suber* and *Fagus sylvatica*). The substantial presence of NLR-ID genes in these species is likely the result of species-specific duplication events involving ancestral NLR-ID genes and/or the recurrent integration of novel IDs into existing NLRs. For example, *Quercus subser*, *Arachis hypogaea* and *Rosa multiflora* presented the greatest diversity of IDs, whereas *Coffea arabica*, *Quercus lobata* and *Quercus subser* presented the highest frequency of a single ID type among angiosperms (Figure S1). The absence of NLR-ID genes in 18 angiosperm genomes could be due to the limited overall count of NLR genes, with the majority of these species (16 out of 18) having fewer than 50 NLRs. A notable exception is *Saccharum* spp. *R570*, which, despite having 168 NLR genes, has no detectable IDs.

To investigate whether the number of NLR-ID genes is correlated with the total NLR number in each species genome, Spearman correlation analysis was conducted. The analysis demonstrated a robust correlation (R = 0.83, *p*-value < 2.2 × 10^−16^) between the abundance of NLR-ID genes and the overall NLR gene count in angiosperm genomes (Figure 1B). The average ratio of NLR-ID genes out of total NLR genes was found to be 10.6%, which is somewhat higher than the previously reported range of 3.5% to 10% based on the analysis of a smaller set of plant genomes [13,19]. Notably, over 34.8% of the angiosperm species analyzed (106 species) presented NLR-ID ratios that exceeded this average. In particular, within the *Brassicaceae* family, all the species presented NLR-ID ratios above 20%, with the exception of *Microthlaspi erraticum* and *Cardamine hirsuta* (Figure S2 and Table S3). Among all the species assessed, the basal dicot species *Boechera retrofracta* had the highest proportion of NLR-ID genes, with an NLR-ID ratio of 38.3% (Figure S2 and Table S3).

To compare the profiles of ID-fusion genes among the three NLR subclasses, we classified NLR-ID genes into CNL-IDs, TNL-IDs, and RNL-IDs, following the established classification system of angiosperm NLR genes [8]. CNL-IDs and TNL-IDs together made up more than 98% of the identified NLR-ID genes, with RNL-IDs representing less than 2% (Figure 1C), mirroring the relative frequencies of these subclasses in angiosperms [26]. However, the proportion of genes containing IDs was notably greater for the TNL subclass than for the CNL and RNL subclasses. Specifically, 5.8% of the CNL and RNL genes were found to be fused with IDs, while this figure reached 29.1% for the TNL subclass (Figure 1C). This disparity suggests a pronounced tendency for TNL genes to incorporate exogenous domains. Upon closer examination of the TNL subclass, we discovered that 80% of the TNL-ID genes contained a specific domain known as the C-JID. When genes containing the C-JID domain were excluded from the analysis, the proportion of TNL genes with IDs decreased to 5.8%, aligning with the rates observed for the CNL and RNL subclasses. The high prevalence of the C-JID domain among TNL-ID genes underscores its potential importance in mediating disease resistance mechanisms. Furthermore, a strong and significant correlation was detected between the number of ID-containing genes and the total number of genes within both the CNL (R = 0.82, *p* < 2.2 × 10^−16^) and TNL (R = 0.95, *p* < 2.2 × 10^−16^) subclasses. In contrast, the RNL subclass exhibited a weaker correlation (R = 0.53, *p* < 2.2 × 10^−16^) (Figure 1B).

### 2.2. Broad Sampling of Angiosperm Genomes Significantly Extends the ID Landscape

**Figure 2:**
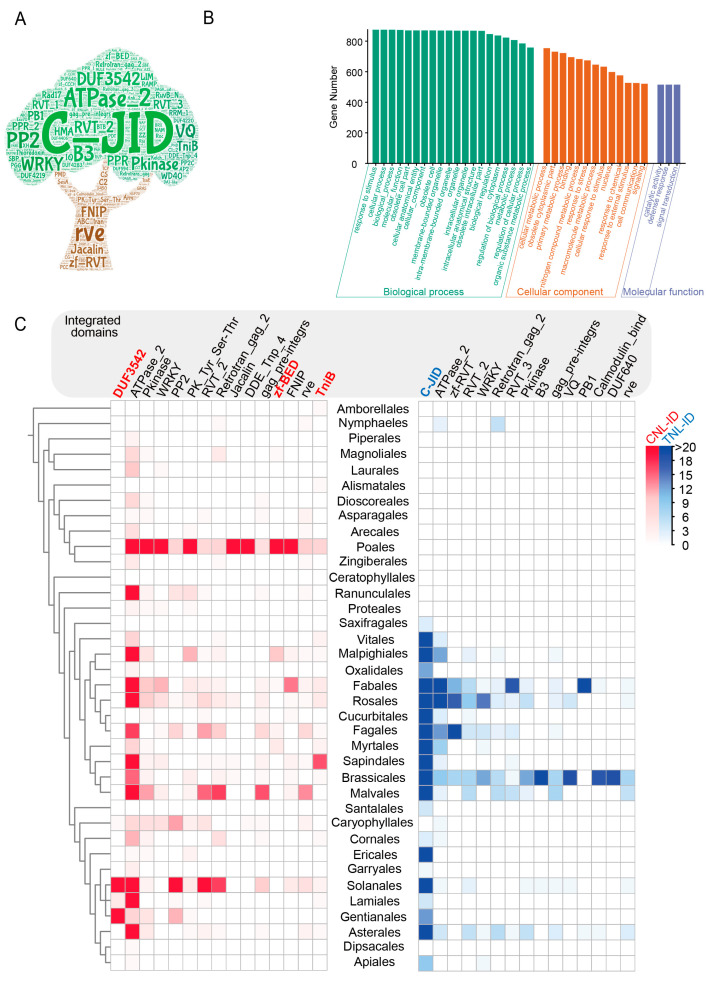
Phylogenetic distribution of IDs across angiosperm species and NLR subclasses. (**A**) The wordcloud of IDs fused to NLRs in 305 angiosperms. (**B**) Gene Ontology functional annotation distribution of IDs fused to the NLR in angiosperms. (**C**) Distribution of the top 15 IDs with the highest frequencies of CNL and TNL in 37 angiosperm orders. Red-labeled IDs are specific to CNLs, and blue-labeled IDs are specific to TNLs. The color gradient indicates the number of NLR-IDs (red represents CNL-ID and blue represents TNL-ID) in each order. The color gradient (covering a range of 0 to >20) indicates the number of NLR-IDs in each order: darker red corresponds to a higher number of CNL-IDs, and darker blue corresponds to a higher number of TNL-IDs.

Our examination of the protein sequences of the 9651 NLR-ID genes revealed a total of 1226 unique IDs (Figure 2A and Table S4). Among these IDs, the C-JID domain was identified as the most prevalent, exhibiting a specific association with TNL proteins. Other IDs frequently found in angiosperm NLR genes include the DUF3542 domain (now renamed the NB-LRR domain in the Pfam database, also known as the Solanaceous domain (SD)) [31], Pkinase domain, WRKY domain, ATPase_2 domain, and RVT_2 domain. Interestingly, 1142 IDs were detected in NLR genes at a low frequency (fewer than ten NLRs), including 678 IDs found in only one NLR gene. These findings suggest that the majority of these IDs may have fused post-speciation, potentially as a means to adapt to specific pathogenic environments.

To explore the potential functions of the identified IDs, we conducted Gene Ontology (GO) analysis. The results revealed a broad spectrum of functions, including signal transduction, defense responses, and enzymatic activities (Figure 2B and Table S5). Additionally, IDs related to retrotransposons, including RVT, Retrotran_gag_2, and zf-RVT, were identified with a high frequency of NLR genes. Although the current evidence does not directly associate these IDs with effector perception by NLR proteins, transposons can contribute to the expansion of NLR genes [32].

To investigate the variation in ID diversity among different NLR subclasses, we conducted a comparative analysis of the ID types found in the CNL and TNL genes. Compared with the TNL genes, the CNL genes included a significantly greater variety of ID types (Figure S3A). To ensure that the observed higher abundance of ID in the CNL gene was not merely a consequence of the relatively lower number of TNL genes in angiosperms, which could be due to the recurrent loss of this NLR subclass across diverse species, we normalized the ID types by the gene number for both NLR subclasses. Even after normalization, the ID diversity remained more pronounced for the CNL genes (Figure S3B). Notably, 200 different IDs were found in both the CNL and TNL genes at the same time, suggesting convergent domain fusion events across the two subclasses (Figure S3A). Further analysis of the 15 most frequent IDs in the CNL and TNL genes across 37 angiosperm orders revealed that only four IDs were exclusively associated with either the CNL (DUF3542, TniB and Zf-bed) or TNL (C-JID) subclasses. By comparison, 11 IDs were found to be common to both subclasses (Figure 2C).

Our analysis of the distribution of different ID types across 37 angiosperm orders revealed several IDs with widespread occurrence. For example, C-JID, Pkinase and ATPase_2 was frequently observed across diverse angiosperm linages, with their presence in 21, 20, and 32 out of 37 orders, respectively (Figure 2C). Conversely, certain IDs, despite their high integration frequency, were found in a limited number of species. For example, the RAMP and DUF4406 IDs were specific to *Durio zibethinus*, and the SH3_19 domain was detected only in *Chrysanthemum seticuspe*. This pattern of limited distribution implies that these IDs may have fused with NLRs post speciation, potentially as a result of recent lineage- or species-specific amplification events (Table S1). These findings collectively indicate that angiosperms have retained ancient NLR-IDs throughout their evolution while also continuously integrating new IDs into NLR genes. This ongoing process of ID incorporation is likely a strategy to adapt to and counteract a diverse range of pathogens encountered during speciation.

### 2.3. A Substantial Proportion of IDs in NLR Genes Are Potentially Targeted by Pathogen Effectors

**Figure 3:**
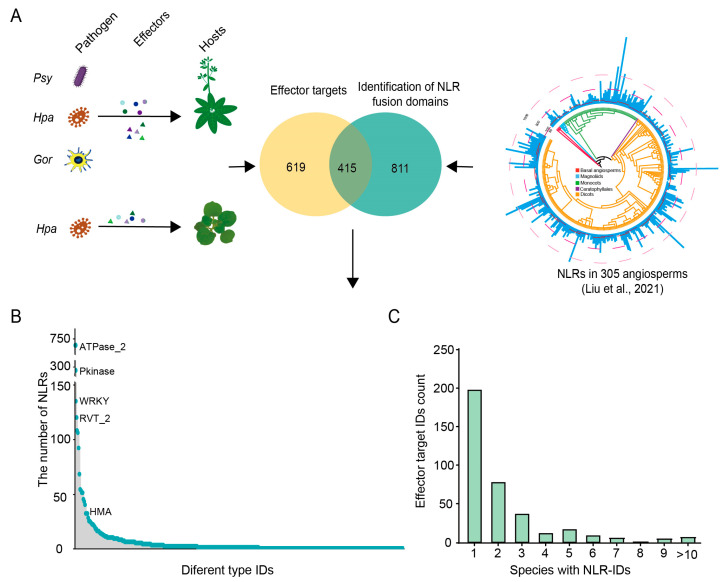
Overlap between IDs and domains present in host proteins targeted by pathogen effectors. (**A**) Overlap between NLR-IDs from this study and functional domains presented in the published “effector interactome”. *Psy*: *Pseudomonas syringae*; *Hpa*: *Hyaloperonospora arabidopsidis*; *Gor*: *Golovinomyces orontii*. The right panel (NLRs in 305 angiosperms) is adapted from Liu et al. [26]. Different shapes (dots, triangles, etc.) represent distinct effectors from pathogens; the arrows represent the process by which pathogens deliver effectors to host plants, and also indicate the association direction between “effector target domains” and “NLR fusion domains”. Downward arrow represents the association between the overlapping region (415 shared domains) of “effector target domains” and “NLR fusion domains” and the subsequent analyses. (**B**) Distribution frequency of overlapping domains fused to NLRs. The lollipop chart (consisting of blue circles and grey bars) represents the number of NLR genes corresponding to each type of ID. (**C**) The histogram indicates the distribution of 415 IDs across 305 species.

The discovery of ID-homologous domains in plant non-NLR proteins targeted by pathogen effectors provides a basis for speculating that these IDs may act as potential mimics of effector targets in the plant-pathogen arms race [11]. Several large-scale studies have identified effector–host protein interactions through yeast two-hybrid or in planta coimmunoprecipitation/tandem mass spectrometry (co-IP/MS) assays in *A. thaliana* and *Nicotiana benthamiana* [27,28,33]. Leveraging these datasets, we conducted an analysis to determine whether the IDs identified in angiosperm NLR genes might serve as a candidate decoy. By investigating the intersection between NLR IDs and domains found in effector-targeting proteins, we found that 415 out of the 1226 IDs were homologous to domains in such proteins of *A. thaliana* and/or *N. benthamiana* (Figure 3A). The frequency of these IDs in NLR genes varied significantly, ranging from 1 to 715 (Figure 3B). Strikingly, 14 IDs were identified as components of *A. thaliana* proteins targeted by three different pathogens, indicating a potential broad-spectrum candidate decoy function (Table S6). Furthermore, 415 IDs were found to be homologous to domains of effector-targeting proteins in *A. thaliana* and/or *N. benthamiana*, suggesting the possibility of a conserved candidate decoy mechanism in plant defense against pathogens.

In fact, several of the IDs that overlap with those domains of effector targets have been reported as decoys or effector-targeting domains of known functional NLR proteins in independent studies (Figure 3B). This includes the HMA domain in the rice CNL proteins RGA5 and Pik-1, which provides specific recognition of the *Magnaporthe oryzae* effectors AVR-Pia and AVR-Pik [18,34,35]. Additionally, the WRKY domain in *Arabidopsis* TNL RRS1 has been identified as an integrated decoy for the effectors AvrRps4 and PopP2 [15,36]. Both the zf-BED and WRKY domains are frequently present in NLR genes (Figure 2C). Moreover, although the roles of certain IDs, such as RIN4, PBS1, and Exo70, remain to be fully elucidated, non-NLR proteins featuring these domains are frequently targeted by pathogen effectors [37,38,39].

The ubiquity and high frequency of certain IDs in NLR genes across angiosperm taxa suggest that these domains may confer broad-spectrum recognition capabilities (Figure 3B,C), potentially serving as candidate decoys for pathogen effectors. On the other hand, nearly half of the IDs that overlap with those targeted by effector proteins are found exclusively in a single angiosperm species, highlighting the evolution of species-specific candidate decoys (Figure 3C). Furthermore, the majority of IDs associated with effector-targeting domains are observed in only a few species, which implies that these domains underwent fusion events later in the evolutionary divergence of species as a strategy to cope with pathogenic challenges. In summary, these observations suggest that many IDs in NLR genes are likely to serve as candidate decoys for pathogen effectors.

### 2.4. ID Fusion and MADA Loss: Unraveling the Evolution of CNL Proteins

**Figure 4:**
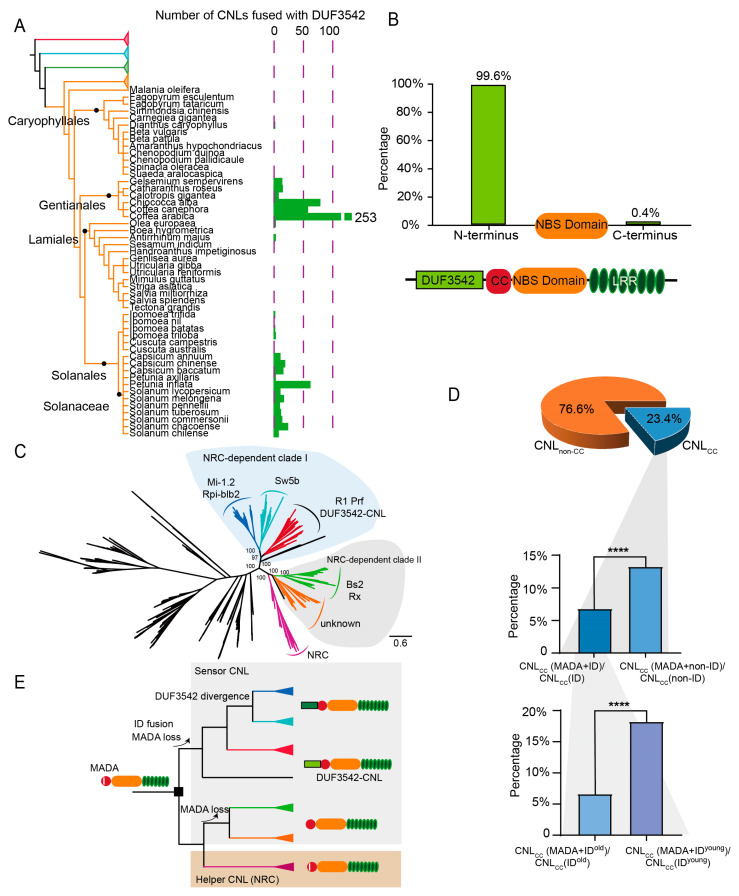
The phylogenetic distribution of the DUF3542 domain in angiosperm CNL genes and its effect on MADA loss. (**A**) Distribution of CNL-DUF3542 in angiosperms. The left panel shows the phylogenetic tree of the super asterid lineage species based on APG IV. The right panel shows the number of CNLs fused with DUF3542. Different colored branches represent different plant lineages: red represent Basal angiosperms, blue represent Magnoliids, green represent Monocots, orange represent Dicots. (**B**) Location of the DUF3542 domain on DUF3542-CNL. The green column represents the proportion of DUF3542 domains located at the N-terminus or C-terminus of DUF3542-CNLs. (**C**) Phylogenetic tree of CNL in Solanaceae. (**D**) Fusion of the DUF3542 domains promoted MADA loss in the CNLs of the NRC clade and NRC-dependent clades. The asterisk indicates a significant difference between the two treatments according to Student’s *t* test (Top panel: *n* = 874 vs. 15690, *p* = 1.4 × 10^−8^, 95%CI = [−8.21%, −4.71%]; Bottom panel: *n* = 864 vs. 114, *p* = 0.0032, 95%CI = [−18.19%, −3.69%]; ****, *p* < 0.0001;). CNL_non-CC_: CNLs without CC domain; CNL_CC_: CNLs with CC domain; CNL_CC_ (MADA + ID)/CNL_CC_(ID): ratio of CNL_CC_ proteins with both MADA motif and ID to total CNL_CC_ proteins containing ID; CNL_CC_ (MADA + non-ID)/CNL_CC_(non-ID): ratio of CNL_CC_ proteins with MADA motif but no ID to total CNL_CC_ proteins without ID; CNL_CC_ (MADA + ID^old^)/CNL_CC_(ID^old^): ratio of CNL_CC_ proteins with MADA motif and ancient ID to total CNL_CC_ proteins with ancient ID; CNL_CC_ (MADA + ID^young^)/CNL_CC_(ID^young^): ratio of CNL_CC_ proteins with MADA motif and young ID to total CNL_CC_ proteins with young ID. (**E**) Loss of the MADA motif and fusion of the DUF3542 domain in the CNLs of the NRC clade and NRC-dependent clades. Different colored lines in (**C**) or triangles in (**E**) represent distinct NRC-dependent clades and NRC clades: dark blue represent Mi-1.2/Rpi-blbl2 clade, light blue represent Sw5b clades, red represent R1 Prf/DUF3542-CNL clades, green represent Bs2/Rx clades, orange represent unknown clades, pink represent NRC clade.

The DUF3542 domain was initially identified within the N-terminus of R1, a CNL protein in potato that confers resistance to late blight [40]. In our study, this domain emerged as the most prevalent CNL-specific ID across 626 NLR proteins. CNL genes with this ID were primarily found in plant species belonging to the *Solanales*, *Gentianales*, *Caryophyllales*, and *Lamiales* orders of the dicot asterid lineage (Figure 4A), with a pronounced expansion in the *Solanales* and *Gentianales* species. Over 99% of the DUF3542 domains identified in NLR genes were fused to the N-terminus of CNL proteins, preceding the CC domain (Figure 4B). A recent study revealed that the DUF3542 domain in a CNL protein (Solyc05g007350) from *Solanum lycopersicum* interacts with the *Ralstonia solanacearum* effector RipAE [23], suggesting a role for the DUF3542 domain as a decoy in the plant immune response. Additionally, mutations in the DUF3542 domain are involved in the enhanced recognition of previously unrecognized TSWV-NSm variants by Sw5b [41]. Phylogenetic analysis of CNL genes from Solanaceous species revealed that the majority of CNL genes containing the DUF3542 domain form a monophyletic clade with the functionally characterized genes *R1* and *Prf* (Figure 4C, Figure S5 and Supplemental Data Set S1). This finding implies that most, if not all, CNL genes with the DUF3542 domain likely originated from a single ancestral fusion event.

The MADA motif within the CC domain is crucial for the immune function of CNL proteins because it allows the formation of pentamer Ca^2+^ channels on the cell membrane [42,43,44]. However, several studies have shown that proteins from the BS2/RX, Mi-1.2/Rpi-blb2, Sw5b, R1/Prf and BS2/RX-sister lineages (Figure 4C) have lost the ability to cause cell death and instead rely on the NRC (NLR required for cell death, a dicot asterid lineage-specific CNL group) proteins to transfer immune signals [9]. Our analysis of the domain structure within the NRC clade and two NRC-dependent clades revealed that the majority of CNLs in the NRC clade retained the MADA motif (Figure 4C and Figure S4). In contrast, all CNL genes in NRC-dependent clades I and II have lost this gene (Figure 4C and Figure S4).

To explore the potential impact of fusion the N-terminal DUF3542 domain or other IDs on the loss of the MADA motif in CNL genes, we analyzed the frequency of the MADA motif in both CNL genes with and without IDs. Among the angiosperm NLR genes classified into the CNL subclass, 23.4% were found to possess an N-terminal CC domain. Upon categorizing these CC domain-containing CNL genes into those without IDs and those with IDs, our analysis revealed a significant difference in the presence of the MADA motif. Specifically, 13% of the CNL genes without IDs contained the MADA motif, whereas only 7% of the CNL-ID genes did (Figure 4D). This pattern is consistent with a model in which ID fusion is associated with accelerated loss of the MADA motif. To further investigate this phenomenon, we compared the proportion of MADA motif-containing genes between “young” CNL-ID genes, which are specific to a single angiosperm species, and “old” CNL-ID genes that are found across multiple species. Compared with the “old” CNL-ID genes, the “young” CNL-ID genes presented a significantly greater proportion of MADA-containing genes (Figure 4D). These results support the notion that ID fusion may be linked to MADA motif loss in CNL proteins (Figure 4E).

### 2.5. Evolutionary Insights into TNL-C-JID Domains and Their Associations with LRR Domains in Angiosperms

**Figure 5:**
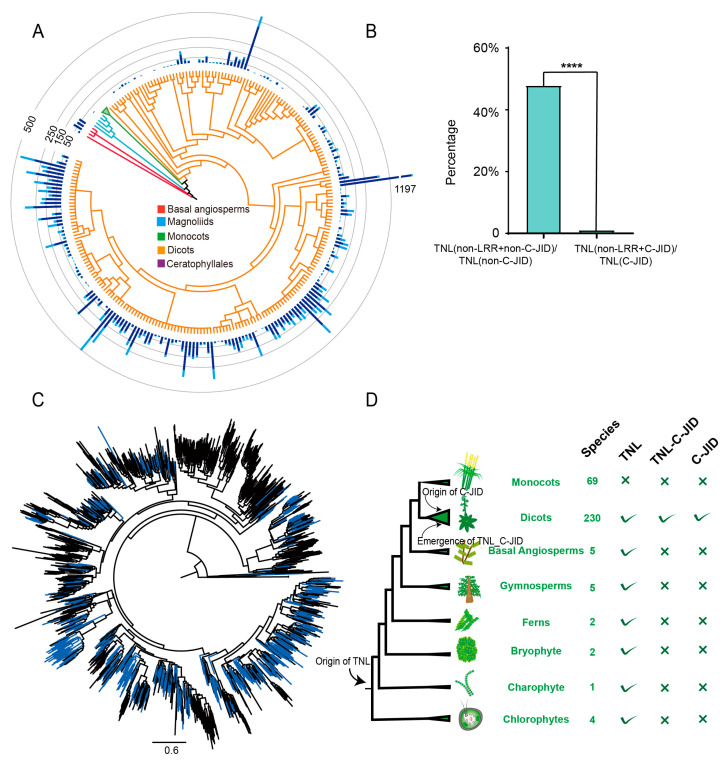
The origin and phylogenetic distribution of the C-JID domain-containing TNL genes. (**A**) Distribution of the C-JID domain in angiosperm species. The column represents the gene number, with light blue representing TNL-C-JID, and dark blue representing TNL-non-C-JID. (**B**) The C-JID domain and LRR domain are closely related. The asterisk indicates a significant difference between the two treatments according to Student’s *t* test (*n* = 14150 vs. 4559, *p* = 1 × 10^−300^, 95% CI = [45.95%, 47.71%]; ****, *p* < 0.0001). TNL (non-LRR + non-C-JID)/TNL (non-C-JID): ratio of TNL proteins with both non-LRR feature and no C-JID to total TNL proteins without C-JID; TNL (non-LRR + C-JID)/TNL(C-JID): ratio of TNL proteins with both non-LRR feature and C-JID to total TNL proteins containing C-JID. (**C**) Phylogenetic tree of TNL in angiosperms. The blue line indicates the TNL fused with the C-JID domain. (**D**) Origin of the TNL-C-JID and C-JID domains. “√ indicates the presence of TNL, TNL-C-JID, or C-JID genes in the plant lineage; × indicates the absence of these genes in the lineage.”.

The C-JID domain was initially identified via structural analysis of two TNL proteins, RPP1 and ROQ1, via cryo-electron microscopy (cryo-EM). This domain is specifically fused to the C-terminus of TNLs, following the LRR domain, and is known to enhance the selective binding of LRR to effectors [45,46,47,48]. The prevalence of C-JID-fusing TNL genes is observed in almost all eudicots that contain TNL genes (Figure 5A). Given the reported function of C-JID in assisting LRR binding to effectors, we investigated whether TNL genes with the C-JID domain are more likely to possess an LRR domain than those without the C-JID domain. Our analysis confirmed this hypothesis, revealing that nearly half of the angiosperm TNL genes lacking the C-JID domain had lost the LRR domain in their protein sequence, whereas only 5.8% of the C-JID-containing TNL genes lacked the LRR domain. This finding also suggests a preference for TNL genes with the LRR domain to undergo fusion with the C-JID domain over those without the LRR domain (Figure 5B).

To elucidate the phylogenetic relationship of TNL genes containing the C-JID domain, we constructed a phylogenetic tree from representative angiosperm TNL genes, which were obtained by clustering the NBS domain of angiosperm TNL genes in the BIG database (PRJCA005581), employing CD-hit with a sequence similarity threshold of 0.6. Our analysis revealed that TNL genes with the C-JID domain are extensively distributed across the angiosperm TNL phylogeny, indicating a pattern of recurrent integration or loss of ancestral integrations (Figure 5C and Supplemental Data Set S2). TNL genes are believed to have originated from the common ancestor of green plants [4,5,6]. However, we did not identify any C-JID domain integrations outside eudicots. Additionally, TNL genes were found to be absent in monocots, and TNL-C-JID was exclusively prevalent in eudicots [46]. These findings suggest that the fusion of TNL genes with the C-JID domain likely occurred after the divergence of the dicot lineage (Figure 5D).

## 3. Discussion

Since the first NLR-type R genes were cloned in 1994, significant progress has been made in understanding the genome-wide organization, sequence diversity, and evolutionary dynamics of NLR genes [49,50,51,52]. Several models of NLR gene recognition and interaction with effectors, including direct interaction, guard, and decoy models, have emerged. The discovery of IDs in several functional NLR genes has led to the development of an integrated decoy model, highlighting an evolutionary adaptation to pathogen pressures through structural innovation. However, three key gaps remained unaddressed prior to this study: (1) the lack of a comprehensive analysis of NLR IDs across representative angiosperm lineages (limiting insights into global ID diversity and distribution); (2) the absence of systematic links between IDs and effector interactomes (hindering efforts to explore their potential decoy-related roles or identify potential effector-interacting proteins); and (3) the unclear evolutionary connections between ID fusion and functional structural features of NLR proteins (e.g., the MADA motif in CNLs). Here, we leveraged the IDs and effector–host interaction datasets to fill these critical gaps.

Recent studies identifying NLR-ID genes from dozens of plant genomes have broadened our understanding of their prevalence [13,19,22,23]. However, many of the IDs from these studies presented only a few species, suggesting that expanded sampling of plant genomes is needed to fully grasp ID abundance and diversity. Recently, an angiosperm NLR atlas was established, which included NLR genes from 305 genomes covering monocots, eudicots, magnoliids, *Ceratophyllales* and early angiosperms [26], allowing for a comprehensive analysis of NLR-ID genes across angiosperms. The detection of NLR-ID genes in 286 angiosperms reinforces the idea that ID fusion is a common strategy for NLR gene evolution. The number of NLR-ID genes (9651) identified in this study accounted for 10.6% of the angiosperm NLR genes, which is slightly greater than the number reported in previous studies in which a small number of genomes were used. However, the proportion of NLR-ID genes varied dramatically among species, ranging from 0 to 38.3%, suggesting that the capacity of the evolving ID of NLR genes can rapidly change during speciation.

Previous investigations have identified more than two hundred different IDs from plant NLR genes [13]. The identification of 1226 different IDs from 9651 NLR-ID genes significantly extends the list of known IDs, with over three-quarters being newly identified. The incorporation of effector-targeting proteins as IDs is hypothesized to be an effective evolutionary path for coping with rapidly evolving pathogens. Consistent with this hypothesis, we observed several domains with widespread distributions across a broad spectrum of species, such as the ATPase_2 domain found in 212 species, the Pkinase domain present in 105 species, and the C-JID domain present in 171 species. Notably, 59 IDs were found in more than 10 species, indicating their potential role as broadly effective immune factors. Furthermore, we identified three IDs, ATPase_2, Pkinase and WRKY, that are present at a relatively high frequency in both the TNL and CNL genes (Figure 2B). The concerted integration of these IDs by different sensor NLR subclasses suggests that these domains may serve as efficient decoys against a variety of pathogens across diverse host plants. This finding is supported by independent studies that revealed that the WRKY, HMA, and Pkinase domains play crucial roles in plant immunity [21,53,54,55].

While numerous IDs are prevalent across a broad range of species, the majority, exceeding 90%, are found in fewer than 10 species. This includes 977 IDs that are restricted to just one or two flowering plant species (Figure S6). Examples of such narrowly distributed IDs include the DUF676 domain, which is specific to *Aegilops tauschii*; the PhoD domain, which is found exclusively in *Arachis hypogaea*; and the RNA helicase domain, which is detected solely in *Boechera retrofracta* and *A. hypogaea*. These instances of species-specific ID fusion events indicate that the “integrated decoy” is not only commonly employed by plants in their defense mechanisms but also an ongoing process throughout plant evolution.

Although the role of IDs as decoys for pathogen effectors is widely hypothesized, direct evidence for this function has been established for only a few instances, such as the WRKY, HMA, DUF761 and BED domains [14,17,18,56]. A recent study surveying the interactome between pathogen effectors and *Arabidopsis* proteins identified 41 out of the 293 IDs as homologs to domains found in effector-targeting proteins [13]. Our comprehensive analysis of the interactome, focusing on the interactions between multiple effectors from *Pseudomonas syringae*, *Hyaloperonospora arabidopsidis*, and *Golovinomyces orontii* with two host plants, *Arabidopsis* and tobacco, revealed that over 400 IDs are homologous to effector-targeting proteins. This accounts for more than one-third of the total number of IDs. Moreover, some of these ID homologous domains identified in *Arabidopsis* have the ability to interact with effectors from multiple pathogens. These findings suggest their high potential as candidate decoys of NLR-ID proteins. Considering that only a few pathogens and host plants were included in the protein interaction analysis, the actual proportion of IDs functioning as decoys is likely greater than our current estimates.

In the zig-zag model of plant-pathogen coevolution, plants detect conserved pathogen-associated molecular patterns (PAMPs) of pathogens through cell surface receptors from the RLK/RLP family to induce PAMP-triggered immunity (PTI), whereas evolved pathogens release effectors into plant cells to block PTI by targeting components of the immune pathway. Under this model, some IDs in NLR genes may act as mimics of plant immune factors, serving as targets for these effectors. In support of this hypothesis, Gene Ontology (GO) analysis revealed that a considerable proportion of the IDs identified in this study are related to plant immunity. The diversity of functional categories among the identified IDs underscores their potential multifunctionality in immune responses. For example, the WRKY domain has been identified as an ID of NLR genes from various angiosperm taxa. Several genes containing the WRKY domain have been implicated in defense against different pathogens, including the TNL genes *RRS1B*, *RRS1-R*, *RRS1-S*, *RRS1-Ws*, and *WRKY19* in *Arabidopsis* against various *P. syringae* strains [13,57,58,59] and the CNL gene *YrU1* from *Triticum urartu* against *Puccinia striiformis* f. sp. *Tritici* [60]. The WRKY family of transcription factors is known for its important and varied roles in plant immune responses, with more than 70% of the WRKY genes in *Arabidopsis* responding to pathogen infection and salicylic acid treatment [61,62]. The CNL protein YrU1, which contains an additional ankyrin ID, is homologous to *Arabidopsis* NPR1 protein, a key regulator in the salicylic acid signaling pathway and a transcriptional coactivator of plant defense responses [60]. Another widespread ID in angiosperm NLR genes is the Pkinase domain. For example, the CNL protein Tsn1 in *T. urartu*, which fuses a Pkinase domain, was reported to function against *Parastagonospora nodorum* [63]. Similarly, the MAPK and RLK family genes containing the Pkinase domain are also well characterized as key signaling components of plant PTI [64,65]. The identification of IDs of NLR genes thus offers an efficient approach for identifying novel immune factors in plants.

The fusion of IDs in sensor NLR genes not only broadens the recognition capabilities but also has implications for their functional mechanisms. Several studies have reported that sensor CNL genes often operate in pairs with different “helper” patterners. In angiosperm asteroid plants, CNL proteins from two closely related lineages require the presence of NRC proteins to elicit immune responses. The function of NRC proteins hinges on a conserved MADA motif, which is crucial for the oligomerization of CNL proteins and the formation of pores in the cell membrane [42]. Interestingly, this motif is entirely absent in genes from NRC-dependent lineages I and II. We hypothesize that ancestral fusion of an N-terminal DUF3542 domain may correlate with the decay of the MADA motif. This fusion may be linked to impaired oligomerization of the CC domain and reduced formation of punching pores on the cell membrane. In support of this assumption, an angiosperm-wide analysis has shown that CNL genes with more ancient fused IDs exhibit a significantly higher rate of MADA motif loss. These results are consistent with a model where ID fusion is associated with MADA motif loss in CNL proteins. Notably, we further analyzed the correlation between the integration of IDs and the length of LRR domains in NLR proteins, and found that the total LRR length of ID-harboring NLRs was significantly longer than that of NLRs without IDs (Figure S7). This observation suggests that IDs and LRRs in this study are not in a functional substitution relationship, and the structural correlation between them awaits further clarification through additional functional validation experiments. In addition, this result complements the strong association between the presence or absence of the LRR domain and the C-JID domain in the TNL subclass (Figure 5B), collectively revealing the coordinated evolutionary characteristics of IDs and core NLR structural domains.

In summary, through an extensive examination of NLR genes across angiosperm genomes, this study substantially extended our understanding of the prevalence and diversity of IDs within NLR genes. Analysis of the pathogen effector–host protein interactome revealed that a significant proportion of IDs could act as decoys for effectors, suggesting a potential role for proteins homologous to these IDs in the immune response of plants. Furthermore, an in-depth investigation of the most frequently fused IDs in both the CNL and TNL genes shed light on their contributions to the functional modalities of sensor NLRs. Although this study advances our understanding of NLR-ID biology with novel insights, it has inherent limitations that offer avenues for future investigation. Most IDs lack experimental functional validation, and host–pathogen coverage is narrow (excluding more plant lineages and pathogen types). Future work could prioritize validating the function of NLR-IDs with high-frequency fused IDs and expand host–pathogen sampling. These findings not only emphasize the importance of IDs in plant NLR evolution and function but also lay a foundation for further exploring ID-mediated immune mechanisms.

## 4. Materials and Methods

### 4.1. Data Sources

The protein sequences of the angiosperm NLR genes utilized in this study were obtained from a previous study [26]. Moreover, the whole-genome protein sequences for non-angiosperm plants were sourced from public databases, as detailed in Table S7.

### 4.2. Protein Domain and Motif Identification

The NLR protein sequences were analyzed via hmmscan against the Pfam-A database (Version: Pfam 33.1; http://pfam.xfam.org/ (accessed on 22 December 2025)). Ref. [66] to identify known domains within the sequences, with an E-value threshold of 0.0001. Given that the Pfam-A database may not be as sensitive when detecting the CC domain, we employed the online conserved domain search (CD-Search) tool (Version: CDD v3.21: https://www.ncbi.nlm.nih.gov/Structure/cdd/wrpsb.cgi (accessed on 22 December 2025)) [67] to specifically identify the CC domain within the NLR proteins. This definition of IDs follows established criteria in previous NLR-ID studies [5,13,19], which similarly define IDs as non-canonical NLR domains (i.e., excluding NBS, LRR, TIR, RPW8, and CC) to maintain consistency with the field’s existing framework. After the removal of typical NLR protein domains, including the NBS, LRR, TIR, RPW8, and CC domains, any remaining domains were classified as integrated domains (IDs) within the NLR proteins. The frequency distribution of these IDs across the 305 angiosperms was visualized via an online word cloud generator accessible at https://wordart.com/create (accessed on 22 December 2025). Furthermore, the MADA motif within the CC domain was identified via HMMER 2.5.1 (http://hmmer.org/) with the hmm profile (elife-49956-supp2-v2.hmm; score > 10) of the MADA motif, which was retrieved from a previous study [42,68].

For the amino acid length of the LRR domain presented in Figure S7, the measurement range was defined as extending from the start position of the first LRR domain to the end position of the last LRR domain within each protein sequence. This sequence segment was verified to contain only LRR domains (with no other structural domains included).

### 4.3. Gene Ontology Annotation of IDs

The amino acid sequences of the IDs of the 9651 NLR-ID genes were subjected to Gene Ontology (GO) annotation via eggNOG-mapper software (Version: eggNOG 5.0; http://eggnog5.embl.de, default parameters) [69]. The GO annotation results were classified and visualized via TBtools-II (Version: v2.138) [70,71,72].

### 4.4. Domain Analysis of Effector-Targeting Proteins of A. thaliana and N. benthamiana

The list of effector-targeting proteins of *A. thaliana* and *N. benthamiana* was retrieved from previous studies [27,28,33]. The sequences of these effector-targeting proteins were subjected to hmmscan analysis against the Pfam-A database to identify domains within each protein [66]. The overlapping IDs and domains within the effector-targeting plant proteins were analyzed and visualized via a Venn diagram.

### 4.5. Phylogenetic Analysis of Representative Angiosperm TNL and Solanaceous CNL

For the phylogenetic analysis of angiosperm TNL genes, the angiosperm TNL protein sequences from a previous study [26] were clustered via CD-hit version 4.6 (threshold:0.6; other parameters set to default values) [73], with an identity cutoff of 0.6. Sequence alignment and phylogenetic analysis of representative angiosperm TNL genes of each cluster were performed as described previously [4], with several CNL sequences as outgroups. Briefly, the amino acid sequences of the NBS domain were aligned via ClustalW 1.82 [74] following the default settings [75] and subsequently manually adjusted via MEGA7 [76]. Genes possessing extremely short or divergent NBS domains were removed from the matrix because of their interference with the precision of the alignment and phylogenetic analysis. Phylogenetic analyses were conducted via the maximum likelihood algorithm with IQ-TREE 1.0 [77]. The best-fit model of amino acid substitution was estimated via ModelFinder [78]. Branch support values were computed via SH-aLRT [79] and UFBoot2 [80] with 1000 bootstrap replicates.

For the phylogenetic analysis of Solanaceous CNL genes, CNL protein sequences were retrieved from 12 Solanaceae genomes as reported in a previous study [26]. To reduce the size of the dataset and the interference of short sequences in the alignment, only CNL sequences with complete NBS motifs (simultaneously possessing the Kinase-2, RNBS-B, GLPL and RNBS-D motifs) were used for further phylogenetic analysis [8]. The procedure used for the sequence alignment and phylogenetic analysis of Solanaceae CNL genes was consistent with that used for angiosperm TNLs described above.

### 4.6. Identification of Genes with DUF3542 and/or C-JID Domains

To determine the distribution of the DUF3542 domain (DUF3542, Pfam: PF12061) and C-JID (C-JID, Pfam: PF20160) across diverse plant genomes, we surveyed the protein sequences of 318 green plant genomes, including 4 chlorophyte, 1 charophyte, 2 bryophyte, 2 fern, 5 gymnosperm and 304 angiosperm plant genomes via the hmmsearch 2.5.1 program [68] with default parameters. All hits were further analyzed via hmmscan in 2.5.1 against a local Pfam-A database (E = 10^−4^) to confirm the presence of the DUF3542 and C-JID domains.

## Data Availability

The original contributions presented in this study are included in the article and its Supplementary Materials (Tables S1–S7, Supplemental Data Set S1 and S2). Further inquiries can be directed to the corresponding authors (zhuqingshao@nju.edu.cn or m18845043187@163.com). Additionally, the protein sequences of angiosperm NLR genes were retrieved from the publicly accessible BIG database (https://ngdc.cncb.ac.cn/omix/releaseList (accessed on 22 December 2025), accession no. PRJCA005581). The whole-genome protein sequences of non-angiosperm plants were sourced from public databases, with detailed access information provided in Supplementary Table S7.

## Supplementary Materials

The following supporting information can be downloaded at: https://www.mdpi.com/article/10.3390/plants15010081/s1. Table S1. NLR-ID genes identified from the 305 angiosperms. Table S2. The number of NLR-ID genes identified across the 305 angiosperm species. Table S3. Proportion of NLR-ID genes in relation to the total NLR gene count and various NLR subclasses for each angiosperm species. Table S4. The frequency of IDs within angiosperm NLR genes. Table S5. GO annotation of angiosperm NLR IDs. Table S6. Domains of pathogen effector targets that overlap with NLR IDs. Table S7. Non-angiosperm genomes used in this study. Supplemental Data Set S1. Maximum likelihood phylogenetic tree of Solanaceae CNL genes (txt). Supplemental Data Set 2. Maximum likelihood phylogenetic tree of angiosperm TNL genes (txt). Figure S1. Number of ID types (left) and the highest frequency ID (right) across angiosperms. The species are arranged on the basis of the number of NLR-IDs within each species. The dashed line represents the average value. Figure S2. Proportion of NLR-ID relative to the total or different NLR subclasses for each angiosperm species. Green column: NLR-ID/NLR; red column: CNL-ID/CNL; blue column: TNL-ID/TNL; light purple column: NRG1-ID/NRG1; deep purple column: ADR1-ID/ADR1. Figure S3. Comparative analysis of ID-containing NLR genes in the TNL and CNL subclasses. A. Venn diagram of ID types fused to CNLs and TNLs. B. Number of ID types in normalized CNLs and TNLs. Figure S4. Phylogenetic tree of CNL in Solanaceae. A circular phylogenetic tree depicting the evolutionary relationships of NLR proteins from various plant lineages. Branches are color-coded by functional lineages: M-1.2/Rp-blb2 (blue), Sw-5b (light blue), R1-PH (red), Bx2/Rx (green), unknown (orange), and NRC (pink). Circular tracks surrounding the tree illustrate domain architectures, including CC (coiled-coil), DUF3542, and other conserved domains. The tree scale (0.5) indicates evolutionary distance. Figure S5. Origin of the DUF3542-CNL gene and DUF3542 domain. Figure S6. Occurrence frequency distributions of different IDs among angiosperm species. Figure S7. Comparison of LRR domain amino acid lengths between CNL and TNL proteins with and without integrated domains (IDs). TNL-non-ID: TNL proteins without ID; CNL-ID: CNL proteins with ID; CNL-non-ID: CNL proteins without ID; the left panel shows the LRR domain length of TNL-ID (non-C-JID) vs. TNL-non-ID; the right panel shows that of CNL-ID vs. CNL-non-ID; **** indicates *p* < 0.0001 (Left panel: *n* = 741 vs. 11404, *p* = 0.000012, 95% CI = [−116.5, −72.61]; Right panel: *n* = 3323 vs. 53741, *p* = 0.000045, 95%CI = [−60.32, −36.41]).
