# Supplementary material for: The Landscape of Integrated Domains of Angiosperm NLR Genes Reveals Continuous Architecture Evolution of Plant Intracellular Immune Receptors
Source: Plants (Basel). 2025 Dec 26;15(1):81. doi: 10.3390/plants15010081 (PMC12787737; doi:10.3390/plants15010081)
Supplement: Supplementary file 1 [file plants-15-00081-s001.zip › Supplementary File/Figure S1.pdf]

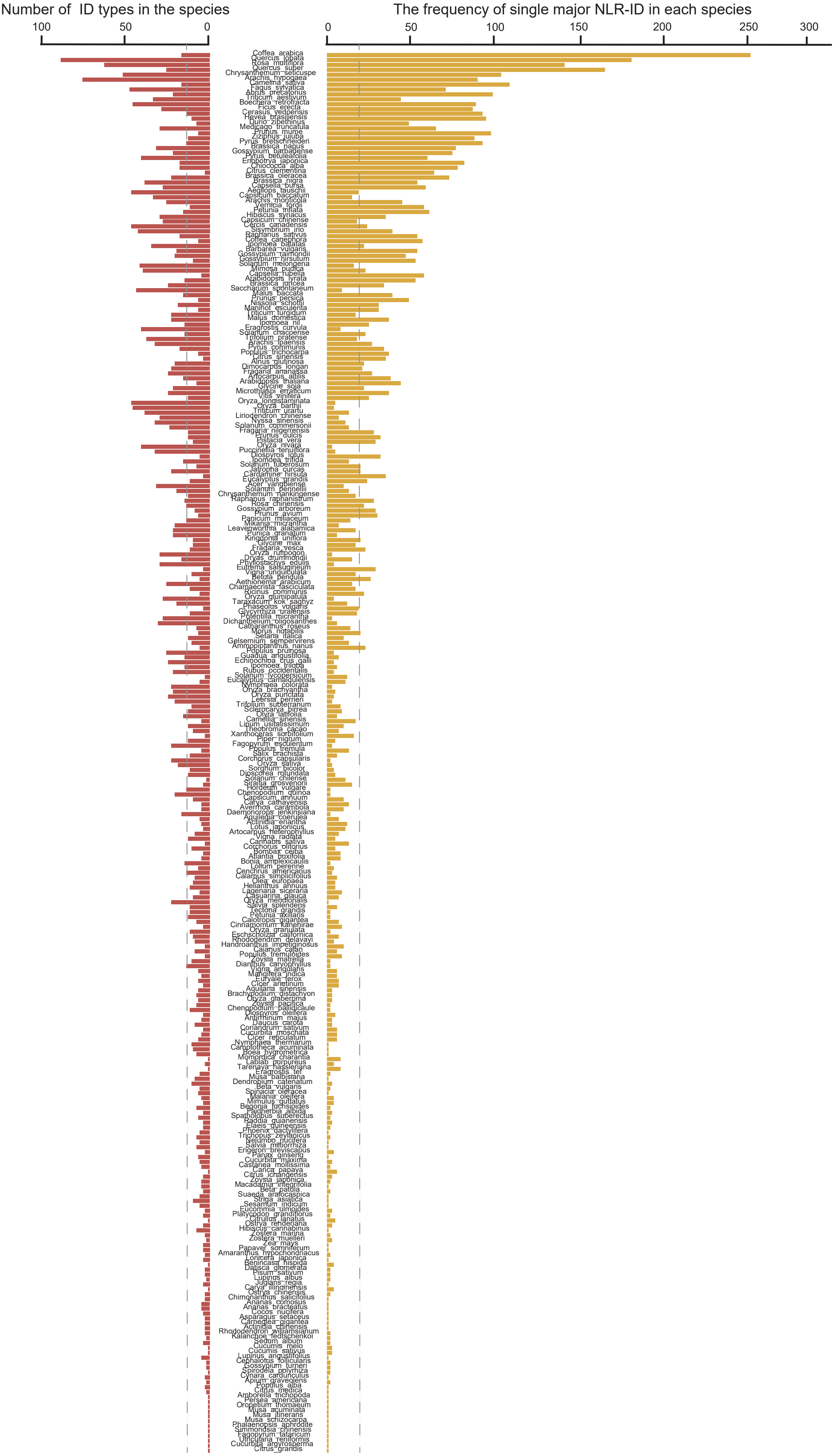

Figure S1. Number of ID types (left) and the highest frequency ID (right) across angiosperms. The species are arranged on the basis of the number of NLR-IDs within each species. The dashed line represents the average value.
