# Supplementary material for: The Landscape of Integrated Domains of Angiosperm NLR Genes Reveals Continuous Architecture Evolution of Plant Intracellular Immune Receptors
Source: Plants (Basel). 2025 Dec 26;15(1):81. doi: 10.3390/plants15010081 (PMC12787737; doi:10.3390/plants15010081)
Supplement: Supplementary file 1 [file plants-15-00081-s001.zip › Supplementary File/Figure S2.pdf]

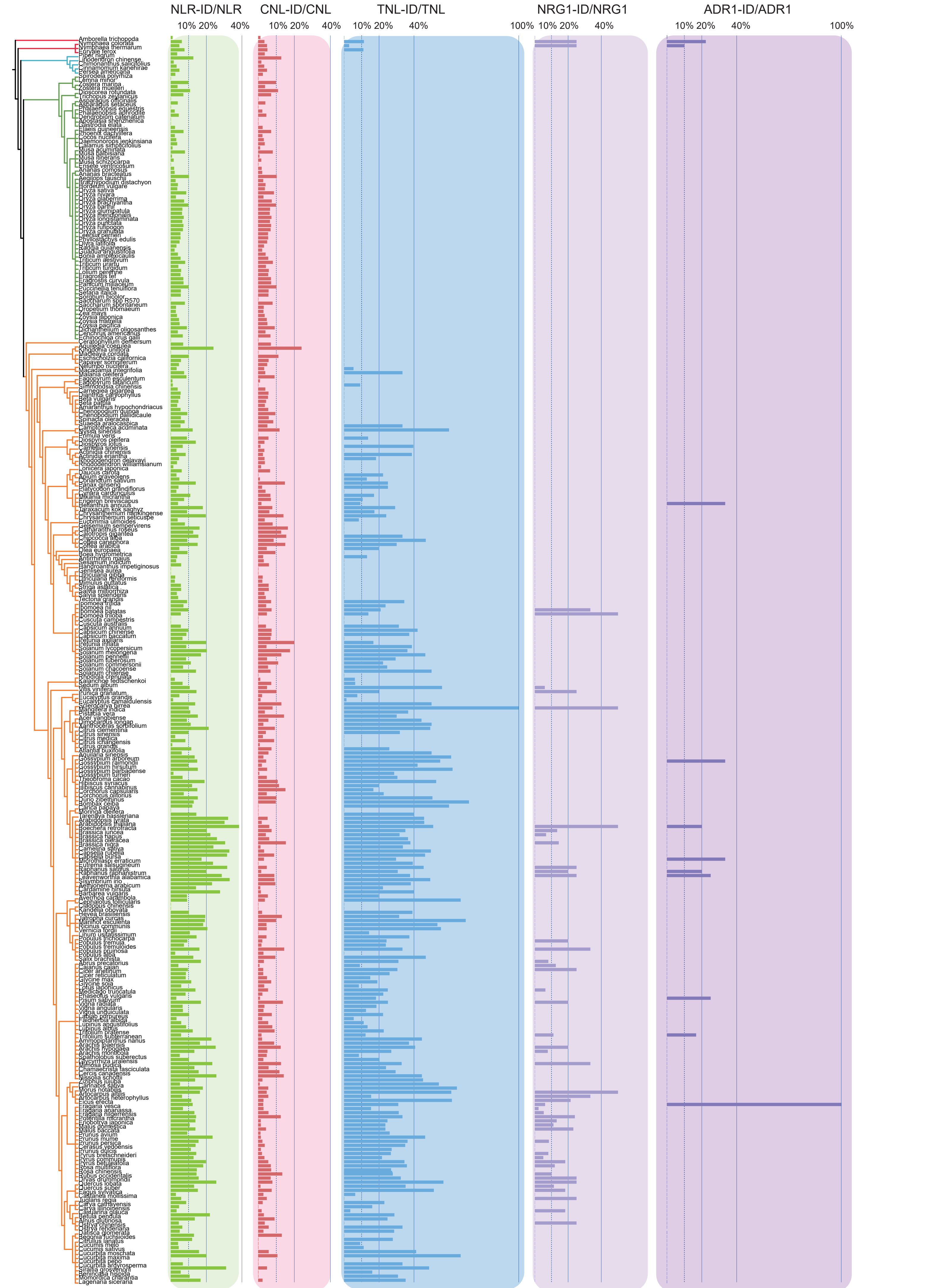

Figure S2. Proportion of NLR-ID relative to the total or different NLR subclasses for each angiosperm species. Green column: NLR-ID/NLR; red column: CNL-ID/CNL; blue column: TNL-ID/TNL; light purple column: NRG1-ID/NRG1; deep purple column: ADR1-ID/ADR1.
