# Supplementary material for: The Landscape of Integrated Domains of Angiosperm NLR Genes Reveals Continuous Architecture Evolution of Plant Intracellular Immune Receptors
Source: Plants (Basel). 2025 Dec 26;15(1):81. doi: 10.3390/plants15010081 (PMC12787737; doi:10.3390/plants15010081)
Supplement: Supplementary file 1 [file plants-15-00081-s001.zip › Supplementary File/Figure S3.pdf]

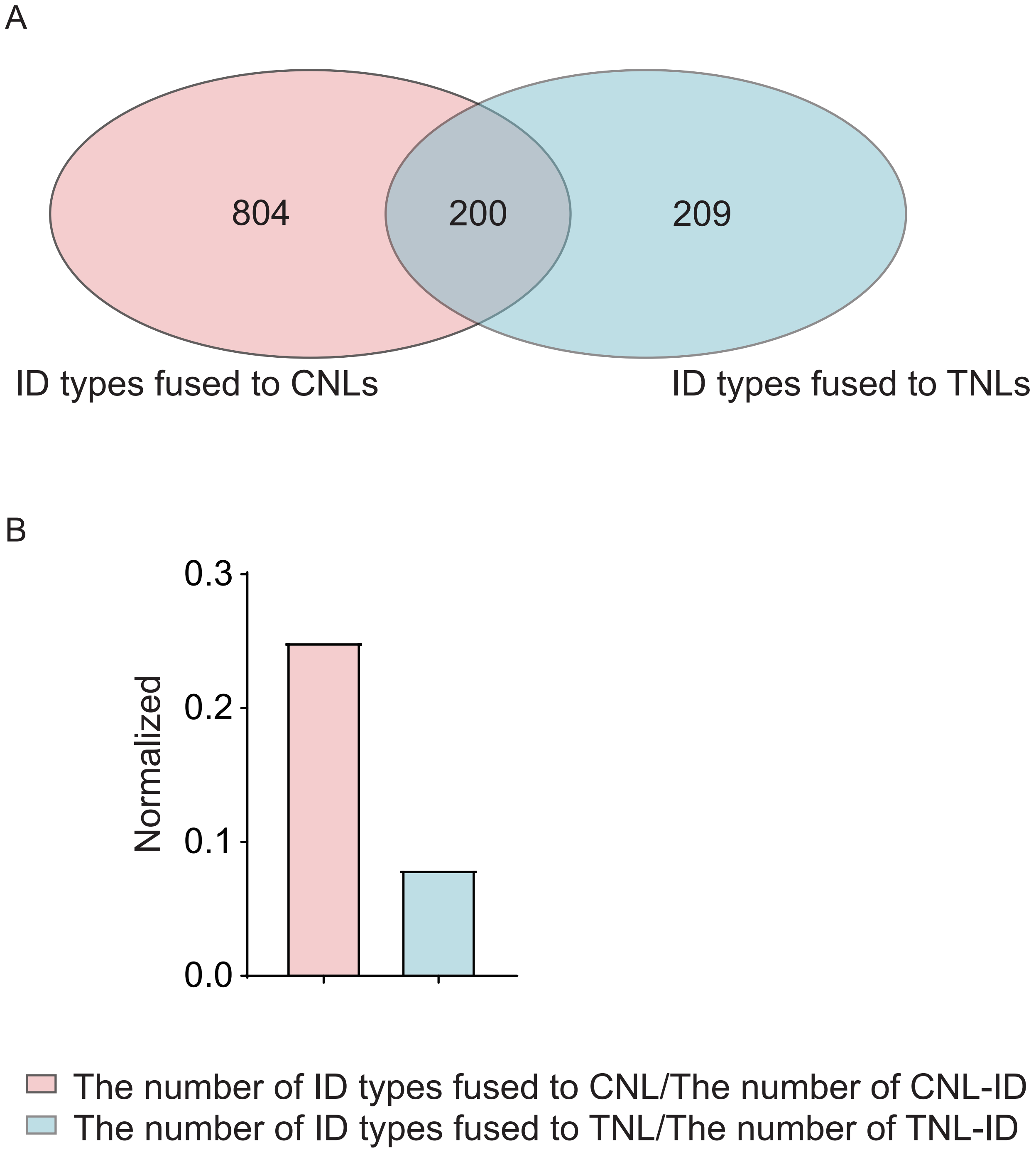

Figure S3. Comparative analysis of ID-containing NLR genes in the TNL and CNL subclasses. A. Venn diagram of ID types fused to CNLs and TNLs. B. Number of ID types in normalized CNLs and TNLs.
