# Supplementary material for: The Landscape of Integrated Domains of Angiosperm NLR Genes Reveals Continuous Architecture Evolution of Plant Intracellular Immune Receptors
Source: Plants (Basel). 2025 Dec 26;15(1):81. doi: 10.3390/plants15010081 (PMC12787737; doi:10.3390/plants15010081)
Supplement: Supplementary file 1 [file plants-15-00081-s001.zip › Supplementary File/Figure S4.pdf]

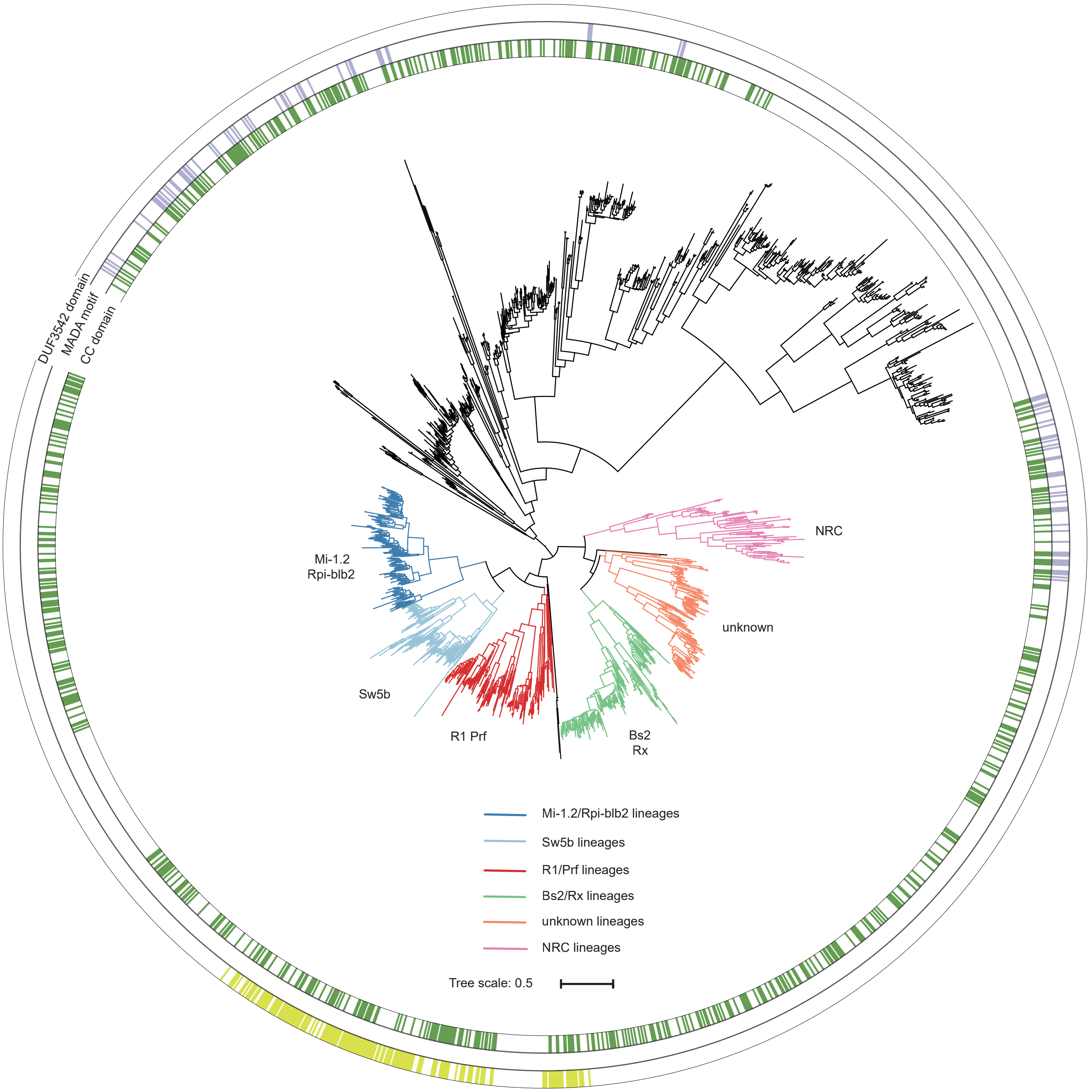

Figure S4. Phylogenetic tree of CNL in Solanaceae. A circular phylogenetic tree depicting the evolutionary relationships of nucleotide-binding leucine-rich repeat (NLR) proteins from various plant lineages. Branches are color-coded by functional lineages: M-1.2/Rp-blb2 (blue), Sw-5b (light blue), R1-PH (red), Bx2/Rx (green), unknown (orange), and NRC (pink). Circular tracks surrounding the tree illustrate domain architectures, including CC (coiled-coil), DUF3542, and other conserved domains. The tree scale (0.5) indicates evolutionary distance.
