# Supplementary material for: The Landscape of Integrated Domains of Angiosperm NLR Genes Reveals Continuous Architecture Evolution of Plant Intracellular Immune Receptors
Source: Plants (Basel). 2025 Dec 26;15(1):81. doi: 10.3390/plants15010081 (PMC12787737; doi:10.3390/plants15010081)
Supplement: Supplementary file 1 [file plants-15-00081-s001.zip › Supplementary File/Figure S7.pdf]

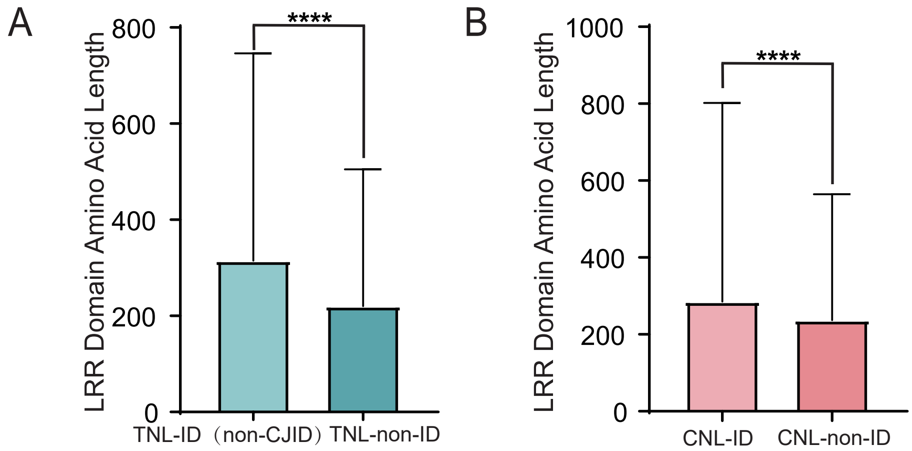

Figure S7. Comparison of LRR domain amino acid lengths between CNL and TNL proteins with and without integrated domains (IDs). TNL-non-ID: TNL proteins without ID; CNL-ID: CNL proteins with ID; CNL-non-ID: CNL proteins without ID; the left panel shows the LRR domain length of TNL-ID (non-C-JID) vs. TNL-non-ID; the right panel shows that of CNL-ID vs. CNL-non-ID; **** indicates $P < 0.0001$(Left panel: n=741 vs 11404, p=0.000012, 95% CI=[-116.5, -72.61]; Right panel: n=3323 vs 53741, p=0.000045, 95%CI=[-60.32, -36.41]).
