# Supplementary figures and images for: The Landscape of Integrated Domains of Angiosperm NLR Genes Reveals Continuous Architecture Evolution of Plant Intracellular Immune Receptors
Source: Plants (Basel). 2025 Dec 26;15(1):81. doi: 10.3390/plants15010081 (PMC12787737; doi:10.3390/plants15010081)

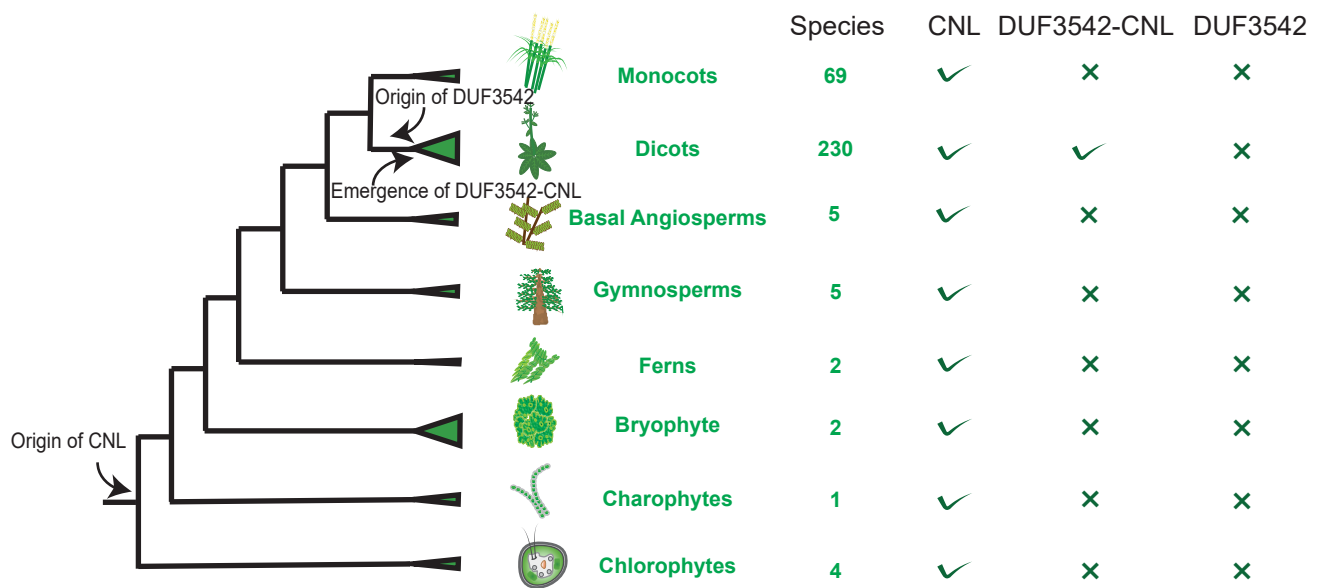

**Figure S5. Origin of the DUF3542-CNL gene and DUF3542 domain.**

Supplement: Supplementary file 1 [file plants-15-00081-s001.zip › Supplementary File/Figure S5.pdf]

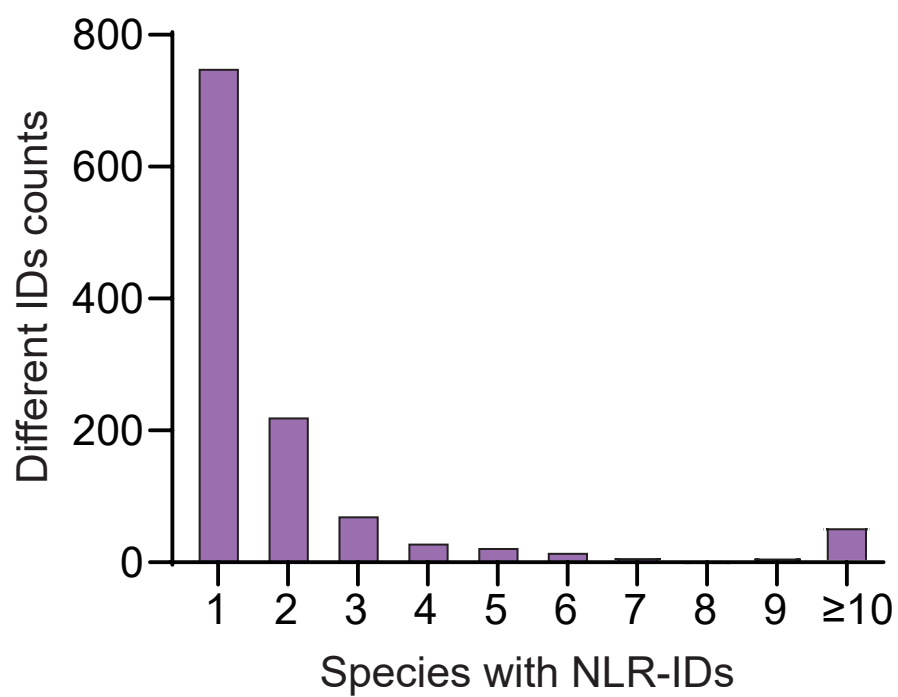

Figure S6. Occurrence frequency distributions of different IDs among angiosperm species.

Supplement: Supplementary file 1 [file plants-15-00081-s001.zip › Supplementary File/Figure S6.pdf]
